# Clonal haematopoiesis and *UBA1* mutations in individuals with biopsy-proven giant cell arteritis and population-based controls

**DOI:** 10.1093/rheumatology/kead435

**Published:** 2023-08-26

**Authors:** Jonas B Salzbrunn, Isabelle A van Zeventer, Aniek O de Graaf, Priscilla Kamphuis, Maaike G J M van Bergen, Yannick van Sleen, Bert A van der Reijden, Jan Jacob Schuringa, Elisabeth Brouwer, Arjan Diepstra, Joop H Jansen, Gerwin Huls

**Affiliations:** Department of Hematology, University of Groningen, University Medical Center Groningen, Groningen, The Netherlands; Department of Hematology, University of Groningen, University Medical Center Groningen, Groningen, The Netherlands; Department of Laboratory Medicine, Laboratory of Hematology, Radboud University Medical Center, Nijmegen, The Netherlands; Department of Hematology, University of Groningen, University Medical Center Groningen, Groningen, The Netherlands; Department of Laboratory Medicine, Laboratory of Hematology, Radboud University Medical Center, Nijmegen, The Netherlands; Vasculitis Expertise Center Groningen, Department of Rheumatology and Clinical Immunology, University of Groningen, University Medical Center Groningen, Groningen, The Netherlands; Department of Laboratory Medicine, Laboratory of Hematology, Radboud University Medical Center, Nijmegen, The Netherlands; Department of Hematology, University of Groningen, University Medical Center Groningen, Groningen, The Netherlands; Vasculitis Expertise Center Groningen, Department of Rheumatology and Clinical Immunology, University of Groningen, University Medical Center Groningen, Groningen, The Netherlands; Department of Pathology and Medical Biology, University of Groningen, University Medical Center Groningen, Groningen, The Netherlands; Department of Laboratory Medicine, Laboratory of Hematology, Radboud University Medical Center, Nijmegen, The Netherlands; Department of Hematology, University of Groningen, University Medical Center Groningen, Groningen, The Netherlands

Rheumatology key messages

- There were no deviating mutational characteristics or larger clonal expansion in 21 biopsy-proven giant cell arteritis cases.

##  

Dear Editor, Somatically acquired and clonally expanded mutations in haematopoietic stem and progenitor cells [clonal haematopoiesis (CH)] may contribute to the pathogenesis of autoimmune diseases, including GCA. CH is observed in 10–40% of individuals ≥60 years of age and has been associated with several inflammation-mediated diseases including atherosclerotic cardiovascular disease, chronic obstructive pulmonary disease and gout. In addition, preclinical murine models show that mutated haematopoietic stem and progenitor cells preferentially expand upon inflammatory stimulation. The potential contribution of CH to the pathogenesis of inflammatory diseases is further exemplified by the presence of *UBA1* mutations in patients with severe inflammatory VEXAS (vacuoles, E1 enzyme, X-linked, autoinflammatory, somatic) syndrome, which can mimic all forms of vasculitis including GCA. Here we questioned whether CH-associated gene mutations, including *UBA1* mutations, contribute to the pathogenesis of GCA and evaluated the clonal expansion of CH over time.

This study was performed within the population-based Lifelines Cohort, a study examining 167 729 persons living in the north of the Netherlands [1, 2]. Details regarding the linkage to the Dutch Nationwide Pathology Databank (PALGA) for the identification of GCA cases, the sequencing methodology for the identification of CH and statistical analysis can be found in the Supplementary Methods (available at *Rheumatology* online) [3, 4]. The Lifelines protocol was approved under number 2007/152, all participants provided written informed consent and the study was conducted in accordance with the Declaration of Helsinki.

Among 152 180 evaluable adult participants from the population-based Lifelines cohort, we identified 100 individuals with a temporal artery biopsy between 1991 and June 2020 (Fig. 1A). After evaluation of the reports by a trained pathologist, 21 individuals with a biopsy-proven diagnosis of GCA were included in the study, together with 84 age- and sex-matched controls that never underwent a temporal artery biopsy. This cohort consists of an admixed population of prevalent and incident cases with GCA [11 individuals had a biopsy performed a median of 3.3 years (range 0–14.4) prior to the baseline assessment and 10 individuals had a biopsy performed a median of 1.5 years (range 0.5–5.3) after the baseline visit]. The annual incidence rate of biopsy-proven GCA was 1.48 (95% CI 0.92, 2.27) per 100 000 individuals ≥50 years of age (Fig. 1B). Interestingly, individuals with an incident diagnosis of GCA had a lower median number of medications used (0 *vs* 3, *P* = 0.004; Supplementary Table S1, available at *Rheumatology* online). This finding is suggestive of a lower burden from comorbidities in individuals prior to a diagnosis of GCA, in line with a recent report of 129 patients [5].

**Figure 1.**
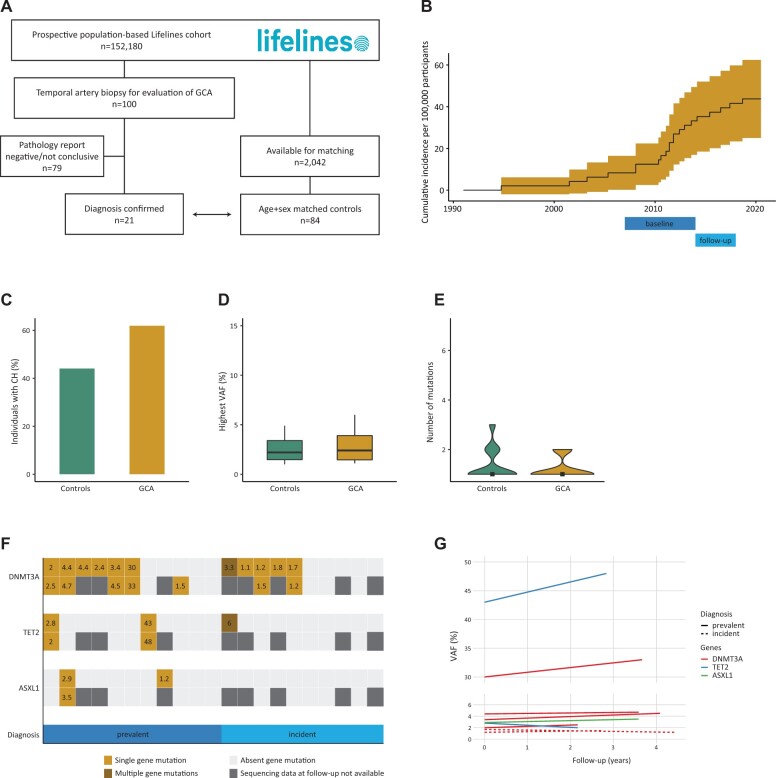
Cohort characteristics and association between GCA and CH. (**A**) Flow diagram showing the selection process of individuals with GCA and the control group. (**B**) Cumulative incidence of GCA with 95% CI for individuals ≥50 years of age. Data reported from the Aalen–Johansen estimator with death as a competing risk. Indicated below is the time interval for baseline and follow-up assessment of the Lifelines Cohort study. (**C–E**) Proportion of individuals with CH (bar plot, chi-squared test), highest observed variant allele frequency (VAF) per individual (boxplot, Mann–Whitney U test) and number of mutations per individual (violin plot, Kruskal–Wallis test). (**F**) Mutational landscape of detected gene mutations at baseline (upper row) and follow-up (lower row) per individual with GCA (vertical columns). The plot is separated for individuals with a prevalent *vs* incident diagnosis of GCA. The highest VAF per gene is indicated. (**G**) Changes in VAF over time for mutations detected at both time points in the cohort of cases with GCA. For the control cohort, see Supplementary Fig. S2 (available at *Rheumatology* online). Expansion of CH mutations over time was estimated by growth rate α = (VAF follow-up/VAF baseline)/time and compared between cases with GCA and the control cohort using the Student’s *t*-test

CH may contribute to the pathogenesis of GCA. In a recent study of 15 patients with GCA, CH was reported in 33% of individuals at the time point of diagnosis [6]. The prevalence of CH is largely dependent on the age of individuals in the cohort and the sequencing methodology that is used for detection of mutations. Here we investigated the prevalence of CH in individuals with GCA from a population-based cohort, which allowed comparison with age- and sex-matched controls from the same cohort. No differences in the prevalence of CH (62% *vs* 44%, *P* = 0.15), number of somatic mutations (*P* = 0.17) or highest variant allele frequency (VAF; *P* = 0.22) were observed between individuals with GCA and their matched controls (Fig. 1C–E). Stratifying the analysis to individuals with a prevalent or an incident diagnosis of GCA did not impact on the results. Among all individuals with GCA we detected mutations in *DNMT3A* in 11 cases, in *TET2* in 3 cases and in *ASXL1* in 2 cases (Fig. 1F, Supplementary Fig. S1, available at *Rheumatology* online). No differences in the prevalence of individual gene mutations between cases with GCA and controls were observed. Longitudinal sequencing data from the baseline and follow-up visit were available for 13 individuals with GCA and 60 controls after a median time period of 3.6 years. The trajectories for individuals with GCA and CH are shown in Fig. 1G and for the control cohort in Supplementary Fig. S2 (available at *Rheumatology* online). There was no difference in the growth rate between individuals with GCA compared with controls (*P* = 0.72). In line with two large-scale sequencing studies, we found no *UBA1* mutations in our cohort of 21 individuals with GCA (13 males and 8 females) or in the cohort of 84 matched controls [7, 8].

The lack of clinical data in this population-based cohort limited our analysis to biopsy-proven cases of GCA. This could have led to an overrepresentation of the cranial subtype of GCA, which may limit the generalizability of our findings to all cases of GCA. Despite this limitation and the overall small cohort size, this is the first study to compare the prevalence of CH and individual gene mutations between individuals with GCA and a matched control cohort and to evaluate the clonal trajectory in time. Our data suggest that the diagnosis of VEXAS is uncommon in patients with GCA and that there is no substantial contribution from the expansion of mutated (immune) cell populations to the pathogenesis of GCA.

## Supplementary Material

kead435_Supplementary_Data

## Data Availability

The article is based on data from the Lifelines Cohort Study. Lifelines adheres to standards for data availability. A request for data access can be sent to the Lifelines research office (research@lifelines.nl). The data catalogue of Lifelines is publicly accessible at www.lifelines.nl.
