# Supplementary material for: Clonal haematopoiesis and UBA1 mutations in individuals with biopsy-proven giant cell arteritis and population-based controls
Source: Rheumatology (Oxford). 2023 Aug 26;63(2):e45–7. doi: 10.1093/rheumatology/kead435 (PMC10836998; doi:10.1093/rheumatology/kead435)
Supplement: kead435_Supplementary_Data [file kead435_supplementary_data.docx]

**Supplementary appendix to**

**Clonal hematopoiesis and UBA1 mutations in individuals with biopsy-proven giant cell arteritis and population-based controls**

Jonas B. Salzbrunn, Isabelle A. van Zeventer, Aniek O. de Graaf, Priscilla Kamphuis, Maaike G.J.M. van Bergen, Yannick van Sleen, Bert A. van der Reijden, Jan Jacob Schuringa, Elisabeth Brouwer, Arjan Diepstra, Joop H. Jansen, Gerwin Huls

Table of Contents

[Supplemental Methods 2](#_Toc139569892)

[Supplemental Table S1 - Baseline characteristics of individuals with GCA compared to age and sex matched controls. 3](#_Toc139569893)

[Supplemental Table S2 - Sequenced genes and regions 4](#_Toc139569894)

[Supplemental Table S3 - Number of identified variants and coverage per gene 5](#_Toc139569895)

[Supplemental Figure S1 - Mutational landscape for the control cohort 6](#_Toc139569896)

[Supplemental Figure S2 - Longitudinal trajectory of CH for the control cohort 7](#_Toc139569897)

[References 8](#_Toc139569898)

# Supplemental Methods

**Study cohort**

The study cohort was selected from the Lifelines cohort. Lifelines is a multi-disciplinary prospective population-based cohort study examining in a unique three-generation design the health and health-related behaviours of 167,729 persons living in the North of the Netherlands. It employs a broad range of investigative procedures in assessing the biomedical, socio-demographic, behavioural, physical and psychological factors which contribute to the health and disease of the general population, with a special focus on multi-morbidity and complex genetics.^1,2^ The baseline assessment of Lifelines took place between 2007 and 2013. For 152,180 participants linkage with the Dutch Nationwide Pathology Databank (Palga) was performed to retrieve all available histopathological reports from temporal artery biopsies. Linkage was based on a pseudonym of the first 8 letters of the last name, initials and the 4-letter postal code. Palga received nationwide coverage in 1991.^3^ Data collection was censored to June 2020. Reports were blinded for outcome variables and manually screened under the supervision of a trained pathologist for a confirmed diagnosis of GCA. Histopathological reports from temporal artery biopsy were retrieved for 100 subjects and out of these 21 reports were identified as conclusive for diagnosis of GCA. Controls were selected from a cohort of 2,042 individuals with available data on CH from targeted next-generation sequencing, that never underwent a temporal artery biopsy. Laboratory assessments and molecular analysis of CH and *UBA1* mutations were performed using peripheral blood samples taken at the Lifelines baseline visit. In addition, peripheral blood from the second screening visit - after a median period of 3.6 years - was sequenced to evaluate the evolution of CH in time. The Lifelines protocol was approved by the University Medical Center Groningen Medical ethical committee under number 2007/152 and the study was performed in accordance with the declaration of Helsinki. All participants provided written informed consent before participating in the study.

**Molecular analysis of clonal hematopoiesis and UBA1**

Error-corrected targeted next-generation sequencing was performed with a custom panel of single-molecule molecular inversion probes (smMIP) on an Illumina NovaSeq 6000 platform, as previously described.^4^ We included 27 common myeloid and lymphoid malignancy-associated driver genes and an additional smMIPs panel targeting the *UBA1* gene (diagnostic for VEXAS) and *PPM1D* (Supplementary Table S2)*.* Somatic variants were called based on a variant allele frequency (VAF) ≥1% and ≥10 consensus variant reads. The mean coverage across all targeted regions was 3044 (Supplementary Table S3).

**Statistical analysis**

The cumulative incidence of GCA was visualized using the Aalen-Johansen estimator, with death as competing risk. Differences in the proportion of individuals with CH, prevalence of individual gene mutations, the highest variant allele frequency (VAF), and number of mutated genes between cases and controls were evaluated by Chi-square, Mann-Whitney U or Kruskal-Wallis tests, as appropriate. Expansion of CH mutations over time was estimated by growth rate $\alpha= (\frac{VAF followup}{VAF baseline})/time$and compared between cases with GCA and the control cohort using the student's t-test. Data was analyzed using R version 4.2.0. Statistical tests were performed two-sided and a p-value < .05 considered significant.

# Supplemental Table S1 - Baseline characteristics of individuals with GCA compared to age and sex matched controls

|  | Control cohort | Biopsy-proven giant cell arteritis | | | | N |
| --- | --- | --- | --- | --- | --- | --- |
|  |  | Prevalent cases | | Incident cases | |  |
|  | N= 84 | N= 11 | P | N= 10 | P |  |
| Age (years) | 70.8 (7.47) | 72.0 (7.62) | 0.63 | 69.5 (7.78) | 0.62 | 105 |
| Male sex (n) | 52 (61.9%) | 7 (63.6%) | 1.00 | 6 (60.0%) | 1.00 | 105 |
|  |  |  |  |  |  |  |
| Total WBC count (10^9^/L) | 5.90 [4.95;6.70] | 5.90 [5.60;9.40] | 0.17 | 4.35 [3.72;5.68] | 0.045 | 105 |
| Neutrophils (10^9^/L) | 3.11 [2.51;3.84] | 3.26 [3.09;3.80] | 0.51 | 2.54 [2.12;3.15] | 0.09 | 101 |
| Basophils (10^9^/L) | 0.03 [0.02;0.04] | 0.03 [0.02;0.04] | 0.65 | 0.03 [0.02;0.04] | 0.92 | 101 |
| Eosinophils (10^9^/L) | 0.17 [0.12;0.31] | 0.11 [0.09;0.15] | 0.12 | 0.15 [0.09;0.22] | 0.41 | 101 |
| Monocytes (10^9^/L) | 0.51 [0.42;0.61] | 0.43 [0.39;0.54] | 0.30 | 0.40 [0.32;0.47] | 0.034 | 101 |
| Lymphocytes (10^9^/L) | 1.82 [1.44;2.30] | 1.90 [1.71;2.20] | 0.47 | 1.33 [1.18;2.24] | 0.25 | 101 |
| Hemoglobin levels (g/dL) | 13.7 [12.4;14.5] | 13.7 [13.0;14.9] | 0.35 | 13.5 [13.0;13.9] | 0.87 | 105 |
| Erythrocytes (10^9^/L) | 4.55 [4.25;4.84] | 4.79 [4.50;4.94] | 0.20 | 4.52 [4.33;4.62] | 0.46 | 105 |
| Hematocrit (L/L) | 0.42 [0.38;0.44] | 0.41 [0.39;0.45] | 0.39 | 0.41 [0.39;0.42] | 0.62 | 105 |
| Platelet count (10^9^/L) | 250 [206;281] | 237 [180;243] | 0.13 | 206 [181;259] | 0.16 | 105 |
| MCV (fL) | 90.7 [87.8;93.7] | 90.8 [87.7;92.1] | 0.57 | 91.6 [90.0;93.3] | 0.67 | 105 |
| CRP (mg/L) | 1.80 [0.70;4.10] | 1.80 [1.70;3.00] | 0.64 | 0.70 [0.60;0.70] | 0.23 | 39 |
|  |  |  |  |  |  |  |
| BMI (kg/m^2^) | 26.2 [24.2;29.0] | 26.5 [24.9;28.5] | 0.71 | 27.7 [25.9;29.3] | 0.51 | 105 |
| HBA1c (mmol/mol) | 39.5 [37.0;41.2] | 39.0 [38.0;45.0] | 0.44 | 41.0 [37.8;42.2] | 0.58 | 89 |
| Medication usage (n) | 3.00 [1.00;5.00] | 3.00 [2.00;5.50] | 0.67 | 0.00 [0.00;2.50] | 0.004 | 105 |

The control cohort is used as a statistical reference group. The number of medications was used as a proxy for comorbidity. Biopsy-proven cases of giant cell arteritis are stratified to incident (diagnosis after baseline assessment) and prevalent cases (diagnosis before baseline assessment); WBC, white blood cell; MCV, mean corpuscular volume; CRP, C-reactive protein, BMI, body mass index; N, number of evaluable individuals.

# Supplemental Table S2 - Sequenced genes and regions

| Gene | Reference transcript | ENSEMBL reference transcript | Exon | Targeted codons/region |
| --- | --- | --- | --- | --- |
| ASXL1 | NM_015338 | ENST00000375687 | 13 (partially) | exon 13 |
| BRAF | NM_004333.4 | ENST00000288602 | 15 (partially) | codon 600 |
| CALR | NM_004343 | ENST00000316448 | 9 | exon 9 |
| CBL | NM_005188 | ENST00000264033 | 8-9 | exon 8 and 9 |
| CSF3R | NM_156039 | ENST00000373103 | 14, 17 | codon 618, 615 and exon 17 |
| DNMT3A | NM_175629 | ENST00000264709 | 2-23 (all coding exons) | all coding exons |
| ETNK1 | NM_018638 | ENST00000266517 | 3 (partially) | codon 243-244 |
| EZH2 | NM_004456 | ENST00000320356 | 2-20 (all coding exons) | all coding exons |
| FLT3_835 | NM_004119 | ENST00000241453 | 20 (partially) | codon 835-842 |
| IDH1 | NM_005896 | ENST00000415913 | 4 (partially) | codon 132 |
| IDH2 | NM_002168 | ENST00000330062 | 4 (partially) | codon 140, 172 |
| JAK2 | NM_004972 | ENST00000381652 | 12, 14 (partially) | codon 617 and exon 12 |
| KIT | NM_000222 | ENST00000288135 | 8 (partially), 17 (partially) | codon 816, 419 |
| KRAS | NM_004985 | ENST00000256078 | 2-3 (partially) | a.o. codon 12, 13, 61 |
| MPL | NM_005373 | ENST00000372470 | 10 (partially) | codon 515, 505 |
| MYD88 | NM_002468.4 | ENST00000417037 | 4-5 (partially) | codon 265 en 232 |
| NOTCH1 | NM_017617.4 | ENST00000277541 | 34 (partially) | codon 2514 |
| NPM1 | NM_002520 | ENST00000517671 | 11 (partially) | codon 288-290 |
| NRAS | NM_002524 | ENST00000369535 | 2-3 (partially) | a.o. codon 12, 13, 61 |
| PPM1D | NM_003620 | ENST00000305921 | 6 | exon 6 |
| RUNX1 | NM_001754 | ENST00000437180 | 2-9 (all coding exons) | all coding exons |
| SETBP1 | NM_015559 | ENST00000282030 | 4 (partially) | codon 850-910 |
| SF3B1 | NM_012433 | ENST00000335508 | 13-16 | codon 575-790 |
| SRSF2 | NM_003016 | ENST00000392485 | 1 (partially) | codon 95, 96 |
| TET2 | NM_001127208 | ENST00000380013 | 3-11 (all coding exons) | all coding exons |
| TP53 | NM_000546 | ENST00000269305 | 2-11 (all coding exons) | all coding exons |
| UBA1 | NM_003334 | ENST00000335972 | 3 | exon 3 |
| U2AF1 | NM_006758 | ENST00000291552 | 2, 6 (partially) | codon 34, 157 |
| WT1 | NM_024426 | ENST00000332351 | 7, 9 | exon 7 en 9 |

# Supplemental Table S3 - Number of identified variants and coverage per gene

| Gene | Number of mutated individuals | | Number of variants | | Mean coverage |
| --- | --- | --- | --- | --- | --- |
|  | GCA | Control | GCA | Control |  |
| ASXL1 | 2 | 3 | 2 | 3 | 2953 (1950-3642) |
| BRAF | 0 | 0 | 0 | 0 | 2544 (1570-3446) |
| CALR | 0 | 0 | 0 | 0 | 4537 (3127-5499) |
| CBL | 0 | 0 | 0 | 0 | 2619 (1525-3398) |
| CSF3R | 0 | 0 | 0 | 0 | 2500 (1628-3227) |
| DNMT3A | 11 | 28 | 12 | 41 | 2914 (1834-3713) |
| ETNK1 | 0 | 0 | 0 | 0 | 5007 (3197-6305) |
| EZH2 | 0 | 0 | 0 | 0 | 3533 (2019-4766) |
| FLT3 | 0 | 0 | 0 | 0 | 4318 (2925-5372) |
| IDH1 | 0 | 0 | 0 | 0 | 4986 (3115-6399) |
| IDH2 | 0 | 0 | 0 | 0 | 2266 (1527-2791) |
| JAK2 | 0 | 1 | 0 | 1 | 2847 (1757-3736) |
| KIT | 0 | 0 | 0 | 0 | 3069 (1821-4095) |
| KRAS | 0 | 1 | 0 | 1 | 1989 (1192-2486) |
| MPL | 0 | 0 | 0 | 0 | 2465 (1751-3146) |
| MYD88 | 0 | 0 | 0 | 0 | 2899 (1540-4175) |
| NOTCH1 | 0 | 0 | 0 | 0 | 370 (166-543) |
| NPM1 | 0 | 0 | 0 | 0 | 850 (497-1149) |
| NRAS | 0 | 0 | 0 | 0 | 3657 (2478-4553) |
| PPM1D | 0 | 4 | 0 | 4 | 2557 (1697-3185) |
| RUNX1 | 0 | 1 | 0 | 1 | 3057 (1705-3948) |
| SETBP1 | 0 | 0 | 0 | 0 | 5214 (3351-6492) |
| SF3B1 | 0 | 0 | 0 | 0 | 2668 (1727-3433) |
| SRSF2 | 0 | 1 | 0 | 1 | 1786 (1169-2334) |
| TET2 | 3 | 10 | 4 | 13 | 3196 (2048-4046) |
| TP53 | 0 | 1 | 0 | 1 | 2706 (1520-3658) |
| U2AF1 | 0 | 1 | 0 | 1 | 2312 (1484-2842) |
| UBA1 | 0 | 0 | 0 | 0 | 1510 (731-2091) |
| WT1 | 0 | 0 | 0 | 0 | 4527 (3022-5638) |

The number of variants per gene and number of mutated individuals for the case and control cohort, using NGS data from the baseline visit. The mean (interquartile range) coverage for each gene across all individuals is given, based on forward and reverse strand.

# Supplemental Figure S1 - Mutational landscape for the control cohort

Mutational landscape of detected gene mutations at baseline (upper row) and follow-up (lower row) per individual (displayed each in a vertical column) for the control cohort. The highest VAF per gene is indicated.

**­­**

**­^

^**

# Supplemental Figure S2 - Longitudinal trajectory of CH for the control cohort

Changes in VAF over time for mutations detected at both timepoints in the control cohort (N=41 mutations in 60 individuals identified)

**

**

# References

1. Scholtens S, Smidt N, Swertz MA, et al. Cohort Profile: LifeLines, a three-generation cohort study and biobank. *Int J Epidemiol*. 2015;44(4):1172-1180.

2. Klijs B, Scholtens S, Mandemakers JJ, Snieder H, Stolk RP, Smidt N. Representativeness of the LifeLines Cohort Study. *PLoS One*. 2015;10(9):e0137203.

3. Casparie M, Tiebosch AT, Burger G, et al. Pathology databanking and biobanking in The Netherlands, a central role for PALGA, the nationwide histopathology and cytopathology data network and archive. *Cell Oncol*. 2007;29(1):19-24.

4. van Zeventer IA, de Graaf AO, Salzbrunn JB, et al. Evolutionary landscape of clonal hematopoiesis in 3,359 individuals from the general population. *Cancer Cell*. 2023;41(6):1017-1031.e1014.
